# Proto-oncogenic miR-744 is upregulated by transcription factor c-Jun via a promoter activation mechanism

**DOI:** 10.18632/oncotarget.11285

**Published:** 2016-08-13

**Authors:** Zhou Sha, Xiaoxia Zhu, Na Li, Yiyi Li, Dianhe Li

**Affiliations:** ^1^ Department of Radiation Oncology, Nanfang Hospital, Southern Medical University, Guangzhou 510515, China

**Keywords:** miR-744, c-Jun, nasopharyngeal carcinoma, non-small cell lung cancer, migration

## Abstract

Upregulation of miR-744 is associated with poor prognosis in many types of cancer patients, but it is still unclear how miR-744 becomes elevated in these tumors. In this study, we found that ectopic c-Jun elevated miR-744 expression, whereas c-Jun attenuation reduced miR-744 expression. Chromatin immunoprecipitation assay confirmed the direct binding of c-Jun to the promoter of miR-744. The binding site of −343 to −349 bp within the most potential promoter like sequence of miR-744 was further validated by luciferase reporter gene assays. C-Jun-induced miR-744 upregulation could significantly promote migration and invasion of nasopharyngeal carcinoma cells and non-small cell lung cancer (NSCLC) cells, hence ectopic c-Jun was sufficient to rescue the migratory and invasive ability of these cells when miR-744 was knockdown. Additionally, a positive correlation between the expression levels of miR-744 and c-Jun was revealed in NSCLC samples with high (top 10%) level of miR-744 expression from the TCGA dataset. Taken together, our results demonstrated for the first time the regulatory mechanism of miR-744 transcription by c-Jun, providing a potential mechanism underlying the upregulation of miR-744 in cancers.

## INTRODUCTION

Cancer metastasis remains a leading cause of treatment failure for cancer patients despite advancements in our understanding of this complex process. Exploration and characterization of genes involved in the initial steps of metastasis, including cell migration and invasion, could lead to novel targets for retarding cancer dissemination.

MicroRNAs (miRNAs) are small, highly conserved non-coding RNAs that have been reported to participate in the metastatic process by negatively or positively regulating the expression of metastasis associated genes through posttranscriptional repression, mRNA degradation, or promoter activation [1, 2]. MiR-744 is identified as a tumor related gene recently, and its dysregulation was found in the serum or specimens from patients with head and neck cancers, multiple myeloma, gastric cancer, hepatocellular carcinoma, or breast cancer [3–7]. We began to notice miR-744 since this gene was found to be remarkably decreased after MTA1 gene knockdown in our previous miRNA microarray analysis [8]. Meanwhile, we also verified the pro-oncogenic potential of MTA1 in nasopharyngeal carcinoma NPC and NSCLC progression and metastasis [9, 10]. These results implied that miR-744 might function as proto-oncogene in NPC and non-small cell lung cancer NSCLC. This hypothesis was recently confirmed by us in human NPC progression and metastasis [11]. However, it has never been reported about the regulatory mechanism of miR-744 expression dysregulation in tumors. Only Sánchez-Jiménez C et al. observed the increase of miR-744 expression in HeLa cells with transient depletion of T-cell intracellular antigen (TIA)-proteins which function as regulators of cell homeostasis [12].

Several mechanisms can control miRNAs expression [13], such as structural genetic alterations, defects in the miRNAs biogenesis machinery, epigenetic changes and so on. An altered transcription factor activity is also one of the important regulatory mechanisms for the deregulation of miRNAs expression. For instance, myogenic transcription factor MyoD could directly bind the miR-182 promoter to increase miR-182 expression hence contribute to cancer progression [14]. Oncoprotein MYC is able to both activate transcription of oncogenic miR-17-92 cluster [15] and directly repress tumor suppressors let-7 [16] and miR-29 family members transcription [17].

In this study, we identified that transcription factor c-Jun directly binds to miR-744 promoter region, resulting in elevated miR-744 expression as well as increased migration and invasion of NPC cells and NSCLC cells. Our data first demonstrated the regulatory mechanism of proto-oncogene miR-744 transcription by c-Jun and uncovered a potential mechanism underlying the upregulation of miR-744 in cancers.

## RESULTS

### MiR-744 transcription is potentially activated by c-Jun

To identify the putative transcription factors (TFs) regulating miR-744 transcription, we used PROMO 2.0 to search for potential transcription factor binding sites (TFBS) in miR-744 promoter region. The bioinformatics analysis revealed the 2-kb region upstream of miR-744 gene contains seven potential binding motifs for transcription factor c-Jun.

**Figure 1:**
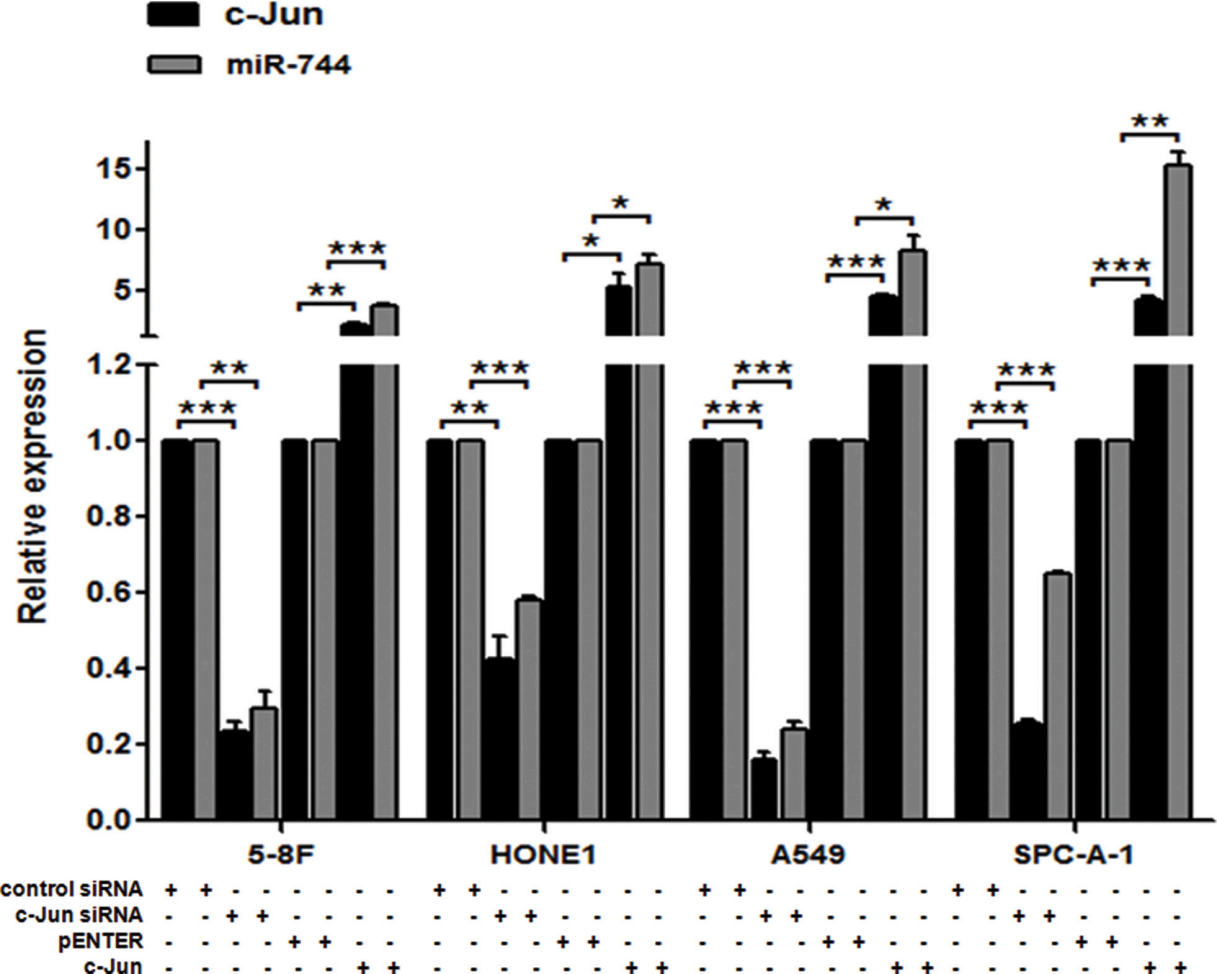
miR-744 transcription is activated by c-Jun qRT-PCR analysis was performed to quantify c-Jun-mediated miR-744 expression in 5-8F, HONE1, A549 and SPC-A-1 cells. GAPDH or U6 was used as an internal control. *P < 0.05; **P < 0.01; ***P < 0.001 compared to controls.

In order to validate the bioinformatical prediction and investigate the critical relationship between c-Jun and miR-744, commercial available c-Jun siRNA, c-Jun plasmid and their controls were used to transiently transfect into 5-8F, HONE1, A549 and SPC-A-1 cells. The transfection efficacy was validated in these transfected cells by qRT-PCR detection for the expression of c-Jun mRNA (Figure 1).

We then examined whether c-Jun knockdown has influence on endogenous expression of miR-744 in these cells. As expected, transfection of c-Jun siRNA, but not control siRNA, led to dramatically reduced miR-744 expression (Figure 1). Meanwhile, overexpression of c-Jun caused a remarkable elevated expression of miR-744 in these cell lines (Figure 1). These results suggested that c-Jun plays a vitally important role in the regulation of miR-744 transcription.

### C-Jun can directly bind to the promoter region of miR-744

In general, TFs can bind to either enhancer or promoter regions of DNA adjacent to the genes that they regulate [18]. To determine whether c-Jun activates miR-744 transcription by directly binding to its putative promoter region as we predicted, a chromatin immunoprecipitation (ChIP) assay coupled with qRT-PCR was performed.

We chose top six potential binding sites for ChIP assay based on the percentage of similarity between c-Jun DNA-binding domains (DBDs) and the predicted c-Jun binding sites in miR-744 putative promoter region, which were −1482 to −1488 bp, −1311 to −1317 bp, −890 to −896 bp, −1261 to −1267 bp, −1582 to −1588 bp and −343 to −349 bp.

Due to the minimum amplicon size for each TFBS is around 150 base pairs in qRT-PCR, it's technically hard to design primers to amplify −1482 to −1488 bp and −1582 to −1588 bp regions separately. Therefore, these two binding sites were amplified as one binding region, renamed to Site 1 in our future study. Similarly, −1261 to −1267 bp together with −1311 to −1317 bp regions were amplified as Site 2 in ChIP assay. The remaining −890 to −896 bp and −343 to −349 bp regions were renamed to Site 3 and Site 4 respectively.

**Figure 2:**
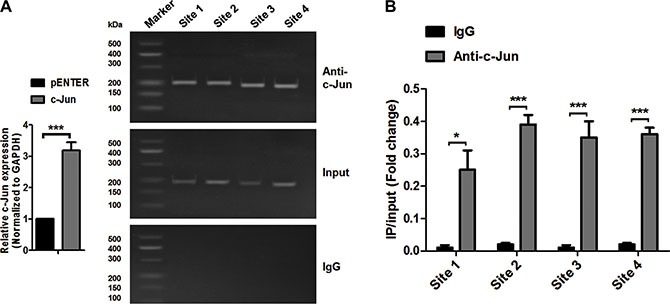
c-Jun can directly bind to the promoter region of miR-744 (**A**) Chromatin immunoprecipitation assay was performed to identify c-Jun binding sites on the miR-744 promoter in A549 cells transfected with a vector expressing c-Jun. An isotype-matched IgG was used as a negative control. (**B**) qRT-PCR analysis was performed with primers specific for 4 binding sites within miR-744 promoter.

As shown in Figure 2A, c-Jun could bind to Site 1, Site 2, Site 3 and Site 4 sequences within the miR-744 putative promoter region in A549 cells, which was validated by quantitative ChIP assays (Figure 2B). An isotype-matched IgG was used as a negative control. These data demonstrated that c-Jun could be recruited to these four binding sites in miR-744 putative promoter region. However, the most functional c-Jun binding site still remains to be characterized.

### The key promoter region of miR-744 contains the c-Jun response element

**Figure 3:**
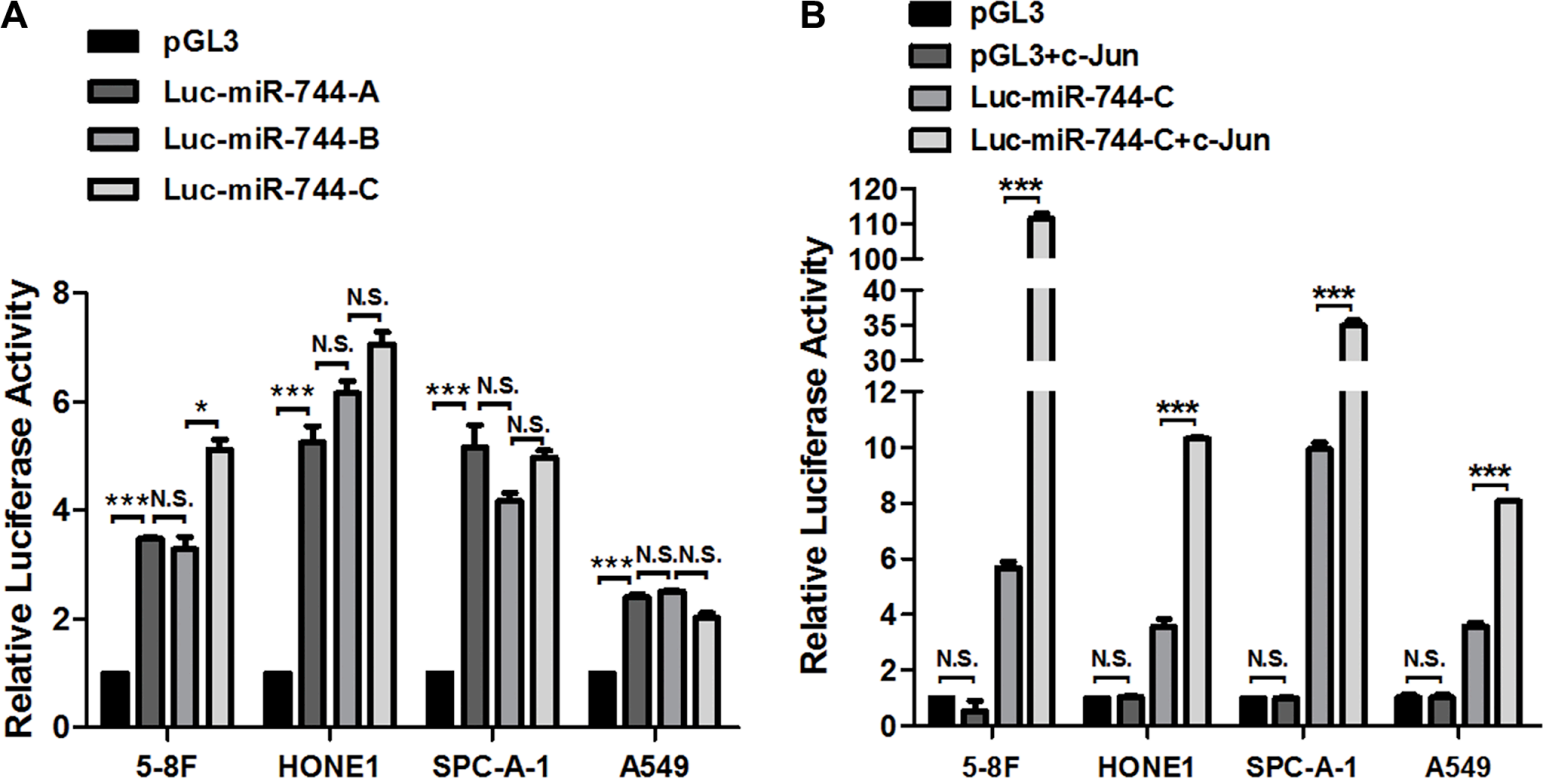
The key promoter region of miR-744 contains c-Jun response element (**A**) Three pGL3-Basic luciferase vector containing deletions of the 5′-flanking region upstream of miR-744 gene were generated. miR-744 promoter A with −2000 bp, miR-744 promoter B with −1000 bp, and miR-744 promoter C with −500 bp upstream of miR-744 gene. Luciferase activity of miR-744 promoter reporter constructs and empty pGL3-basic vectors were measured in 5-8F, HONE1, SPC-A-1 and A549 cells. (**B**) Luciferase activity of miR-744 promoter C reporter construct and empty pGL3-basic vectors in the presence of c-Jun plasmids or its vector control were measured in 5-8F, HONE1, SPC-A-1 and A549 cells. *P < 0.05; **P < 0.01; ***P < 0.001 compared to controls. N.S., non-significant.

To determine the most potential promoter region of miR-744, we cloned −2000 to −1 bp of 5′-flanking region upstream of miR-744 gene into pGL3-reporter vector upstream of the luciferase gene (named as miR-744 promoter A) and conducted a dual luciferase assay in 5-8F, HONE1, SPC-A-1 and A549 cells. Insertion of this region resulted in 2-fold to 5-fold upregulation of relative luciferase activity. To further screen for the most potential promoter like sequence, we created another two sequence deletion versions of the promoter constructs ranging from −1000 to −1 bp of miR-744 gene. Both constructs with −1000 bp (named as miR-744 promoter B) and with −500 bp (named as miR-744 promoter C) upstream of miR-744 gene maintained a similar promoter activity as compared with putative full promoter of −2000 bp, indicating a most potential promoter like sequence in −500 to −1 bp region (Figure 3A).

To validate the existence of the c-Jun response element in this key promoter region of miR-744, we performed co-transfection studies. The luciferase activity were detected in 5-8F, HONE1, SPC-A-1 and A549 cells transfected with both miR-744 promoter C (-500 to −1 bp) or empty pGL3-basic vector and c-Jun plasmid or its corresponding empty vector control for 48 h (Figure 3B). Consistently, miR-744 promoter construct with only −500 bp caused a significant increase of relative luciferase activity in 5-8F, HONE1, SPC-A-1 and A549 cells, and this is further enhanced by c-Jun by 2 to 18-fold, suggesting a functional promoter activated by c-Jun within the −500 to −1 bp region upstream of miR-744 gene.

### C-Jun enhances the promoter activity of miR-744 by directly binding to its promoter

Since the -500 to -1 bp region was validated to be the key promoter region of miR-744 and be responsive to c-Jun, furthermore, the bind of c-jun with the putative binding motif in the -343 to -349 bp region (Site 4) upstream of miR-744 gene was demonstrated by a ChIP analysis of c-Jun-treated A549 cells, this binding site was chosen for further experiment to validate promoter activation of miR-744 by c-Jun through directly binding to its promoter.

**Figure 4:**
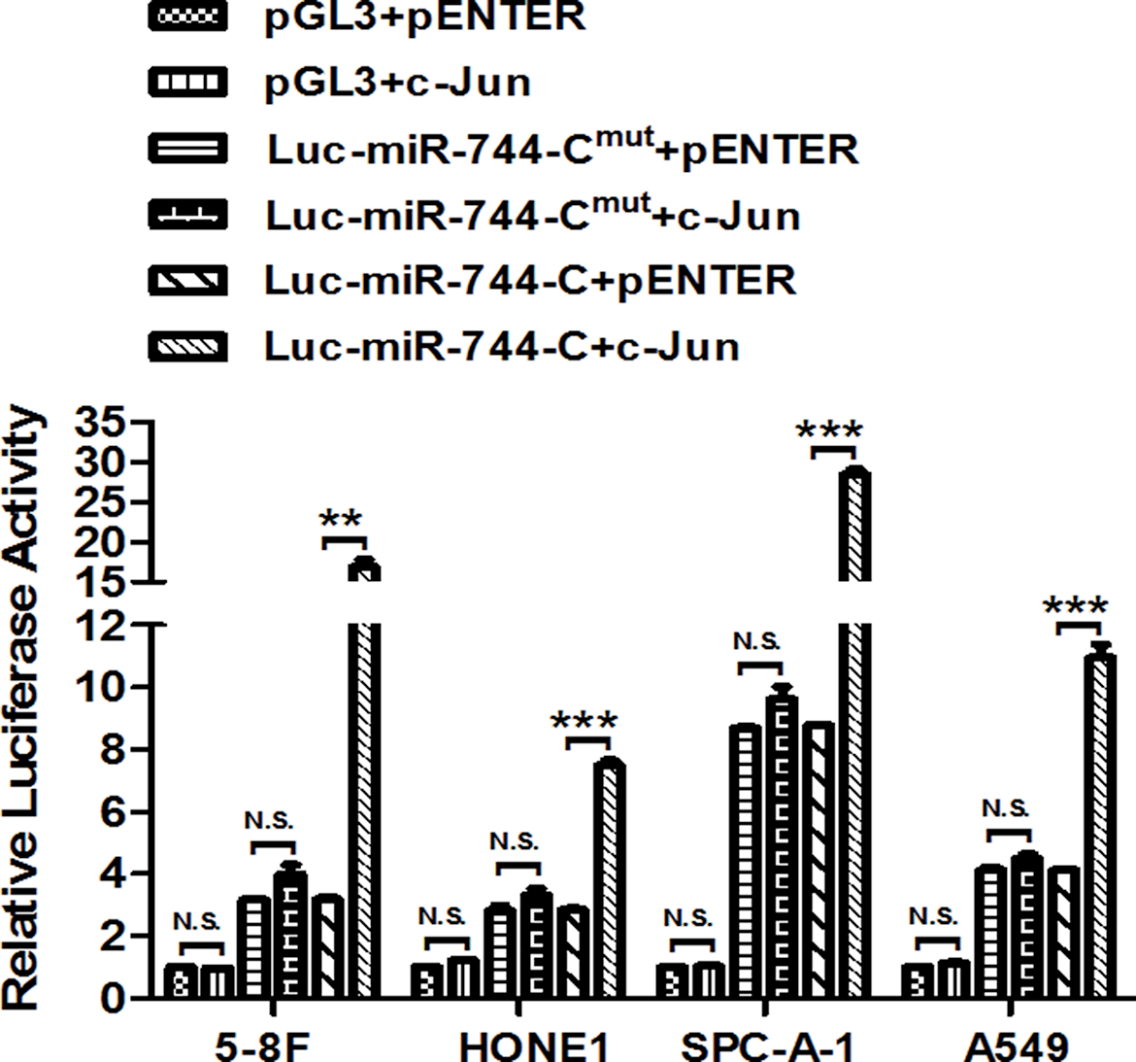
c-Jun enhances the promoter activity of miR-744 by directly binding to its promoter MiR-744 promoter C activity was measured by performing luciferase reporter assay upon mutation of the c-Jun-binding motif (MUT) in the presence of c-Jun plasmids or its vector control in 5-8F, HONE1, SPC-A-1 and A549 cells. **P < 0.01; ***P < 0.001 compared to controls. N.S., non-significant.

The identified c-Jun-binding motif Site 4 was converted to the complementary sequence to eliminate potential c-Jun binding by using a site-direct mutagenesis approach (Supplementary Table S3). Ectopic c-Jun significantly increased the luciferase activity of firefly reporter gene fused with wild-type c-Jun-binding motif Site 4, whereas mutation of the motif completely abrogated c-Jun-enhanced promoter activity in 5-8F, HONE1, SPC-A-1 and A549 cells (Figure 4). All these results strongly suggested that the direct bind of c-Jun with binding motif Site 4 in miR-744 promoter enhances the transcription of miR-744 by activating its promoter.

### MiR-744 mediates the promotion of cancer cells migration and invasion by c-Jun

**Figure 5:**
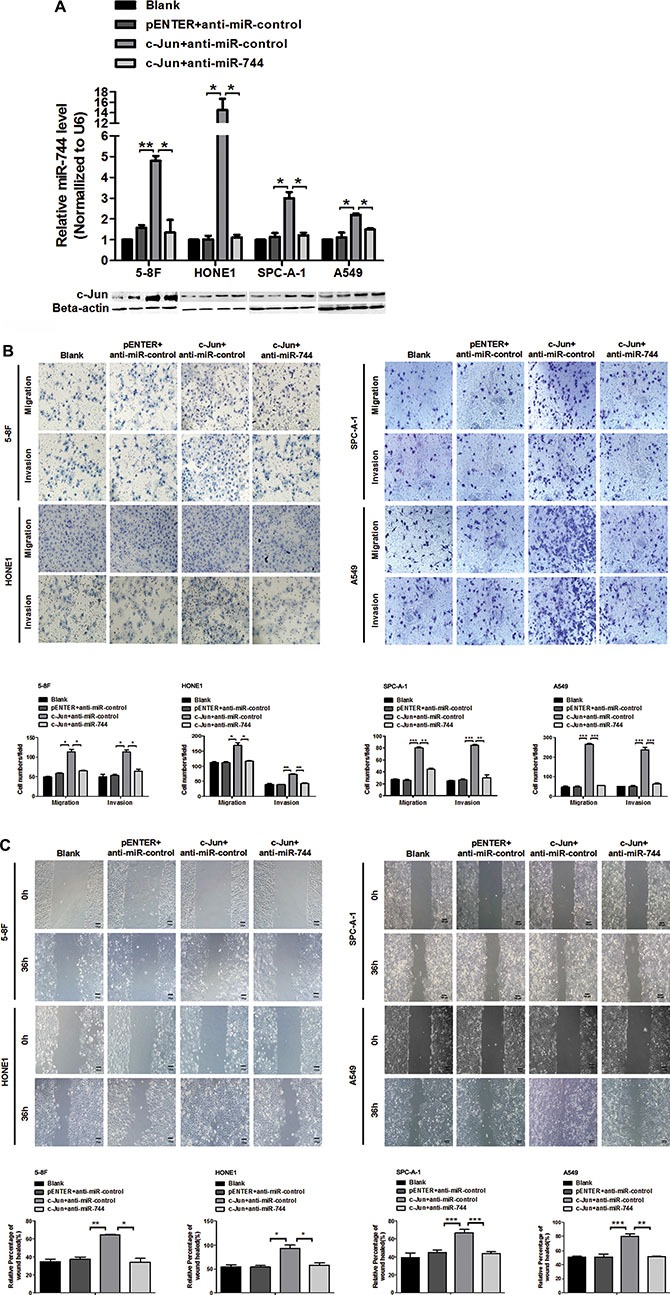
miR-744 mediates the c-Jun promotive effects on cancer cells migration and invasion (**A**) Expression of miR-744 and c-Jun were analyzed by qRT-PCR and western blot respectively in 5-8F, HONE1, SPC-A-1 and A549 cells co-transfected with miR-744 inhibitor or its vector control and c-Jun plasmids. (**B**, **C**) C-Jun promoted the motility and invasion of 5-8F, HONE1, SPC-A-1 and A549 cells. MiR-744 inhibitor almost completely attenuated this positive effect of c-Jun. The cells motile and invasive activities were measured using (B) Transwell, Boyden and (C) wound healing assays, respectively. Original magnification: 200×. *P < 0.05; **P < 0.01; ***P < 0.001 compared to controls. N.S., non-significant.

It has been shown that c-Jun promotes an aggressive phenotype in cancer cells, including increased migration and invasion [19]. Also, we previously reported that overexpression of miR-744 enhances the migratory and invasive ability of cancer cells [11]. To characterize whether c-Jun induced cell biological behaviors could be mediated by miR-744, we performed co-transfection of c-Jun plasmid and miR-744 inhibitor in 5-8F, HONE1, SPC-A-1 and A549 cells. Enforced expression of c-Jun was confirmed by western blot (Figure 5A). qRT-PCR showed that c-Jun overexpression led to a significant increase of miR-744 expression as compared with control cells (Figure 5A). Interestingly, decreased miR-744 by miR-744 inhibitor could even be rescued to normal level by c-Jun in these cells (Figure 5A).

We then examined whether c-Jun caused promotion of cancer cell migration and invasion could be counteracted by miR-744 inhibitor. In scratch assay, transwell migration and invasion assay, we observed that overexpression of c-Jun significantly promoted 5-8F, HONE1, SPC-A-1 and A549 cells migration and invasion (Figure 5B and 5C), whereas the positive effect of c-Jun treatment on migration and invasion was almost completely attenuated by miR-744 inhibitor. These data made it obvious that miR-744 expression is increased by c-Jun thus involving in the promoting effects of c-Jun on tumor migration and invasion.

### Correlations between c-Jun and miR-744 levels in NSCLC tissues

**Figure 6:**
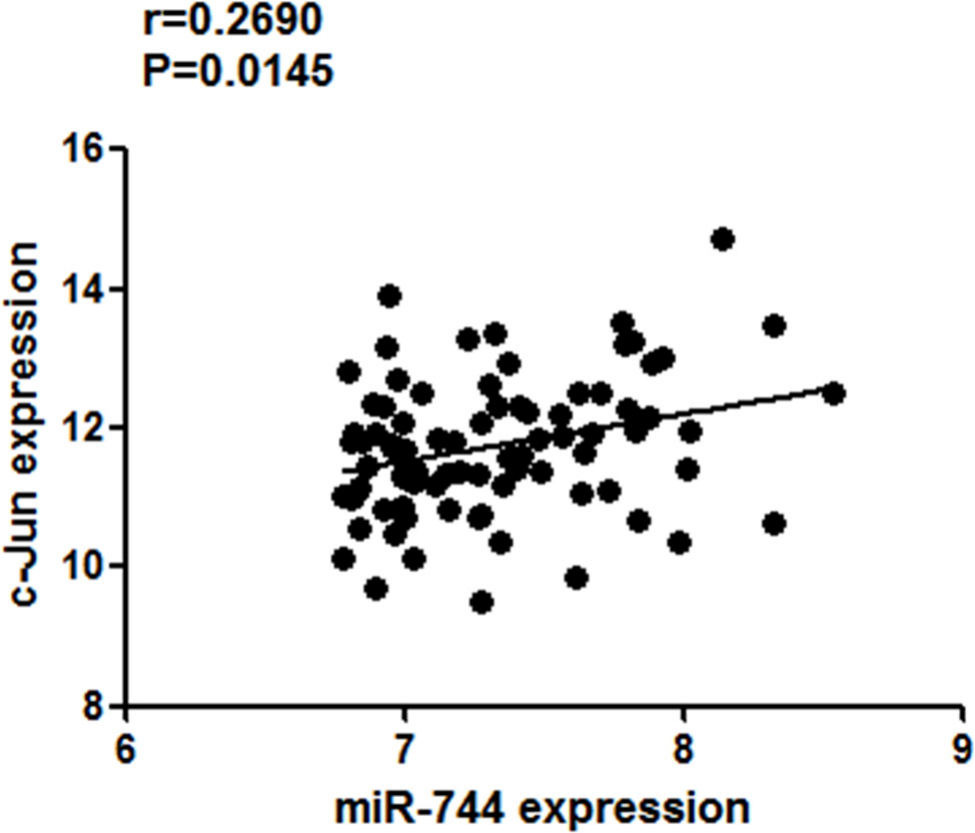
Association of miR-744 expression with c-Jun mRNA levels in NSCLC from TCGA dataset A positive correlation between miR-744 and c-Jun levels was presented in NSCLC samples (*n* = 82) with high (top 10%) level of miR-744 expression.

The mRNA-seq and miRNA-seq data for lung adenocarcinoma and squamous carcinoma were downloaded from TCGA websites (https://tcga-data.nci.nih.gov/maintenance_deploying.html). Both miR-744 and c-Jun expression were retrieved from the datasets. The mRNA and miRNA expression data were processed level 3 data (RNA-seq V2). The samples were sorted from high to low according to the level of miR-744 expression, and top 50%, 40%, 30%, 20%, 10% of cases were taken respectively. Only from 82 samples (top 10% of cases), a positive correlation between the expression levels of miR-744 and c-Jun was revealed (*r* = 0.2690; *P* = 0.0145; Figure 6). The results implied that c-Jun may partially contribute to the upregulation of miR-744 expression in NSCLC.

## DISCUSSION

Although differential levels of miR-744 dysregulation have been observed in many types of cancer, the regulatory mechanism underlying these dysregulation remains unknown. According to the data from the UCSC Genome Browser (http://genome.ucsc.edu/), miR-744 is an intronic miRNA and MAP2K4 is its host gene. Generally, an intronic miRNA is transcribed via the promoter of its host gene [20]. However, it's been proven that intronic miRNAs can also be independently transcribed from their host gene via their own promoter positioned immediately upstream of miRNAs [21]. Here, we identify a most potential promoter like sequence located within 500 bp upstream of miR-744 gene, which implied the existence of a promoter of miR-744 independent of its host gene MAP2K4.

A deregulation of miRNA expression can be a result of epigenetic changes in the promoter of miRNAs, structural genetic alterations in miRNA genes, defects in the miRNA processing, or altered transcription factor activity [13]. The upmodulation of oncogenic miRNAs in cancer can be due to aberrant histone deacetylation, DNA hypomethylation, genetic amplification, gain-of-function mutation, or increased oncogenic transcription factor activity [13]. As the first discovered oncogenic transcription factor [22], c-Jun has been proven to be associated with cancer initiation and progression. C-Jun can not only form the AP-1 early response transcription factor in combination with c-Fos, but also act as an independent transcription factor [23–25]. In the current study, we have shown the first demonstration that transcription factor c-Jun plays an important role in miR-744 transcriptional upregulation. MiR-744 can be directly induced by the tumor promoter c-Jun and be partially responsible of the invasive phenotype induced by this oncogene. We also identified -343 to -349 bp upstream of miR-744 gene as the direct and efficient c-Jun binding site in miR-744 promoter region. Our data revealed for the first time that miR-744 as a tumor-promoting gene could be increased by c-Jun in NPC cells and NSCLC cells, while the regulatory mechanism of miR-744 as a tumor suppressor in other types of cancers still remains to be clarified.

Finally, we detected the correlation of miR-744 and c-Jun levels in NSCLC samples from TCGA. A positive correlation was presented only in NSCLC with high (top 10%) level of miR-744 expression, which proved that c-Jun partially contributes to the upregulation of miR-744 expression in NSCLC.

Taken together, the data herein provide for the first time the mechanistic insights into the miR-744 dysregulation and establish a direct link between c-Jun signaling and miRNAs during this dysregulation. Progress in elucidating the mechanisms for the upregulation of miR-744 expression in cancers establishes the theoretical basis to determine when and how anti-miR-744-targeted therapy may be feasible. The present mechanism offers a new insight into the oncogenic functions of miR-744 and a better understanding of the miR-744 upregulation in cancer. C-Jun-induced upregulation of miR-744 mediates the effects of c-Jun signaling in NPC and NSCLC. Further studies on the role and mechanism of miR-744 in NSCLC will be summarized in our next study.

## MATERIALS AND METHODS

### Cell lines and cell culture

The 5-8F, HONE1, SPC-A-1 and A549 cells were purchased from Shanghai Cell Bank of Chinese Academy of Science (Shanghai, China) and all cultured in RPMI-1640 medium (Corning, NY, USA) supplemented with 10% fetal bovine serum (FBS, GIBCO, South America, NY, USA). All these cells were maintained with 5% CO_2_ atmosphere at 37°C.

### Plasmid and oligonucleotide construction

Based on miRbase database (http://www.miRbase.org), the miR-744 inhibitor (anti-miR-744) and its negative control miRNA (anti-miR-control) were designed and synthesized by Genepharma (Shanghai, China), together with siRNA against c-Jun and its non-specific siRNA control. Plasmids expressing c-Jun and its negative control pENTER were designed and synthesized by Vigene Bioscience (Shandong, China).

### Transient transfection

The 5-8F, HONE1, SPC-A-1 and A549 cells were seeded in 6-well plates at 30–40% density. Transient transfection was performed with Lipofectamine 3000 reagents according to the manufacturer's instructions (Invitrogen, USA). For all the experiments, cells were collected at 24 h or 48 h after transfection.

### RNA extraction and quantitative real-time PCR (qRT-PCR) analyses

Total RNA was extracted from cell lines by using TRIzol^®^ Reagent (Invitrogen, CA, USA) according to the manufacturer's instruction. For the detection of mature miR-744, total RNA was reverse transcribed into cDNA and then used to perform qRT-PCR by using SYBR^®^ PrimeScript™ miRNA RT-PCR Kit (TaKaRa, Dalian, China). For the detection of c-Jun mRNA, total RNA was reverse transcribed into cDNA by using PrimeScript™ RT reagent Kit (TaKaRa, Dalian, China). The quantitative real time PCR for c-Jun mRNA was performed on Mx3005P real-time PCR instrument (Stratagene, USA) by using SYBR^®^ Premix Ex Taq™ (TaKaRa, Dalian, China). Small nuclear RNA U6 (U6 snRNA) or Glyceraldehyde-3-phosphate dehydrogenase (GAPDH) was used as an internal control for miRNA or mRNA quantification respectively. The sequences of the primers used for qRT-PCR were listed in Supplementary Table S1.

### Western blotting

Equal amounts of proteins were separated by 10% SDS-PAGE gels and blotted onto nitrocellulose membranes. The blots were incubated with primary antibodies against c-Jun (1:1000; Abcam, Cambridge, USA) and β-actin (1:2000; Santa Cruz Biotechnology, CA, USA) at 4°C overnight. The membranes were incubated for 2 h with horseradish peroxidase-conjugated goat anti-mouse secondary antibody (1:5000; Santa Cruz Biotechnology, CA, USA) for 1 h at room temperature. β-actin was used as a loading control.

### Chromatin immunoprecipitation assay

ChIP assay was performed with EZ-ChIP Kit (Milipore, USA) according to the manufacturer's instructions. Cells were suspended in lysis buffer and then sonicated on ice to shear the chromatin into length of 500 bp to 1 kb in average. The lysates were diluted using ChIP-dilution buffer (0.01% SDS; 2 mM EDTA; 1% Triton X-100; 150 mM NaCl; 20 mM Tris-HCl, pH 8.0; and protease inhibitors). Anti-c-Jun antibody (ab31419, 1:250; Abcam) or normal rabbit IgG (negative control, 1:500; Abcam) were added to immunoprecipitate the supernatant and incubated overnight at 4°C. Protein G-agarose was added to precipitate the immunocomplex, the beads were washed and eluted with elution buffer (0.1 M NaHCO;1% SDS; and 200 mM NaCl). Reversal of crosslinking was performed with NaCl by heating at 65°C for 6 h. After this step, RNase A was added at 37°C to digest the RNA contaminants for 0.5 h. Samples were treated with 0.5 M EDTA, 1 M Tris-HCl, Proteinase K for 2 h at 45°C, and the DNA was purified with a spin column. Finally, quantitative real-time PCR was performed using the DNA isolated, with the primers provided in Supplementary Table S2.

### Promoter reporter construction and luciferase assays

Different length of the upstream regions of miR-744 gene were amplified with PCR. Mutagenic primers were designed for the c-Jun-binding motif within -349/-343 with 7 bp mismatch. The amplified products were then cloned into the pGL3-basic vector to generate miR-744 promoter constructs. The sequences of these promoter constructs were listed in Supplementary Table S3. All the constructs were confirmed by DNA sequencing (HuaAnPingKang Co., Ltd., Shenzhen, China). The 5-8F, HONE1, SPC-A-1 and A549 cells were plated onto 24-well plates at a density of 5 × 10^3^ cells/well ahead of transfection study. 100 ng of miR-744 promoter constructs or empty pGL3-basic vector were transfected into these cells using Lipofectamine 3000 and incubated for 48 h. For co-transfection, 100 ng of miR-744 promoter C or empty pGL3-basic vector and 1 ug of c-Jun plasmids or its corresponding empty vector control were used. Dual luciferase reporter assay system (Promega, WI, USA) was used to perform the luciferase assay according to the manufacturer's instruction.

### Cell migration and invasion assays

Cell invasion assay was assessed by using transwell chamber (Corning, NY, USA) in 24-well plate. Each group of cells (10^5^ cells/100 μl) was resuspended in serum-free medium and seeded onto the upper chamber with 5% matrigel-coated membrane (BD Bioscience, San Jose, CA) at 48 h post-transfection, while the lower chamber was filled with 500 μl fresh complete medium. After incubated for 24 h at 37°C with 5% CO_2_, non-invading cells were removed from the upper surface of the filter by scraping with a cotton swab. Invading cells that adhered to the lower surface of the chambers were fixed in methyl alcohol and stained with hematoxylin. The invading cells were manually counted at 200× magnification in three random fields by using inverted microscope. Similar inserts without matrigel were used to perform the migration assay.

### Wound healing assay

When cells were grown to approximately 90% confluency (after 48 h of transfection), an artificial wound was created with a 20 μl pipette tip. The cells were then cultured in fresh medium. To visualize wound healing, images were taken at 0 h and 36 h. The relative percentage of wound healed was calculated as (the width of wound at 0 h - the width of wound at 36 h)/the width of wound at 0 h.

### Statistical analysis

Data are expressed as the mean ± standard error of triplicates. Each experiment was repeated at least three times. Statistical analyses were performed using SPSS 13.0 (SPSS Inc., Chicago, USA). The student's *t*-test was used to assess differences among groups in *in vitro* studies. In all cases, *P* < 0.05 was considered to be statistically significant.

## SUPPLEMENTARY MATERIALS TABLES




