# Supplementary material for: Proto-oncogenic miR-744 is upregulated by transcription factor c-Jun via a promoter activation mechanism
Source: Oncotarget. 2016 Aug 13;7(40):64977–86. doi: 10.18632/oncotarget.11285 (PMC5323131; doi:10.18632/oncotarget.11285)
Supplement: Supplementary file 1 [file oncotarget-07-64977-s001.pdf]

Proto-oncogenic miR-744 is upregulated by transcription factor c-Jun via a promoter activation mechanism

Supplementary Materials

Supplementary Table S1: Gene-specific primers for quantitative real-time PCR

GAPDH	F: GCACCGTCAAGGCTGAGAAC<br>R: TGGTGAAGACGCCAGTGGA
U6	F: GCTTCGGCAGCACATATACTAAAAT<br>R: CTCAACTGGTGTCGTGGA
miR-744	F: ACACTCCAGCTGGGTGCGGGGCTAGGGCTAAC<br>R: CTCAACTGGTGTCGTGGA
c-Jun	F: TCCAAGTGCCGAAAAAGGAAG<br>R: CGAGTTCTGAGCTTTCAAGGT

Supplementary Table S2: Specific primers for quantitative real-time PCR in ChIP assay

Site 1	F: TGGAAAAATTGCTTCCCAAT<br>R: CTGCCATTATTTGCCCACTT
Site 2	F: AATCCCGTTGAAGCTGTGTC<br>R: GGGAAGCAATTTTTCCACAT
Site 3	F: TTTGCAGCTCTTGGGATTCT<br>R: GACACAGCTTCAACGGGAT
Site 4	F: GTGGGCAAATAATGGCAGTT<br>R: ACACCCTCAAAGGAGTTGGA

Supplementary Table S3: The sequences of the promoter constructs in promoter activity assay. See Supplementary_Table_S3
