# Supplementary material for: Proto-oncogenic miR-744 is upregulated by transcription factor c-Jun via a promoter activation mechanism
Source: Oncotarget. 2016 Aug 13;7(40):64977–86. doi: 10.18632/oncotarget.11285 (PMC5323131; doi:10.18632/oncotarget.11285)
Supplement: Supplementary file 2 [file oncotarget-07-64977-s002.docx]

**Supplementary Table 3**

| The sequences of the promoter constructs in promoter activity assay | |
| --- | --- |
| Luc-miR-744-A | tgccactgatttccgggcttcttgtgattctggatgctagagtatcaaataatatgggtgaaaggactttgaactgaaaagcacttgtatgaatgaaagcttatcacttagtatgatttgaaagtctgttatttggaacactgatttagtaaattgttatttagaccaagtgtatgaatctttgaagtgtaactttgaaccttaatattagtacatacgattattctcagagtgggggtagattttaaagttctaagttttttgggaaccttaaaggttgctacagaaaaccattctactaattggagaaaactttcctaaattttggaagaagaaaaataatgaccaagcacataaactttgcttgtttgcatcataggatcagagtttaaaagagatcctgatcatttagtatgaaactgaaaaatgtagttgatatttaatgaattctttcattgtgtgtatggttgatagtttgttcctacacattggggtttggtgtgtgtgtgagagagagcaagagagagaaacagattgattttacatttttcattggatttacttttttacaaagcatggttagaagttattaatactttgatctccaggttgagattttggagatgattttttatttaatggaaatatgtaatgagtgagctcttttgtctacgagccactcattttttatcccattgctttatcgaacgagggctgaaaatttcaattttgaatagcacatggcaattttcctcttagagccagctagctattcagttaactgttctcccacctcaggatggtattaacaagaacaggacagtatactaaggcactagttcccaaacattactggaatcatctgggggtctttaaaaatactgttttctgactcctgtccctggataattcagatgtaattgctatgggacgatacctgggcattgagatttttaaaatctcccccaggtgattgtaaggtacagcaaaccttgggaatagttgtactaactgaagattgaggcactggaataactaatgttgggtaagatagggtaagaactttttcgcaataaggtttgcagctcttgggattctcacttgataatgctgtttggataaaccatcatccaagtttgaataagatcttatatttagaaatgaaaactgatctgcagaattagtttaatcaataaatgggaatttttaaaaatcttcttggcaatttgtttttatgaatcccgttgaagctgtgtctattgactacctaaatctagatcattcttaatccagtgtgtaaaaaaccaggatgacacaaatgaaaaacttcaaaaacctggaggtcagactattttagtaatttagaaaacatttttcccacacattaatcagtactaaaagaaaaaagttaaaacctatttaaaatgtggaaaaattgcttcccaatattttaacagagagagactgagaacacacagcattgagtcatcaggaaaactgaagatctcccctgaacaacactgggatttcactgcagaggacttgaaagaccttggagaaattggacgaggagcttatggttctgtcaacaaaatggtccacaaaccaagtgggcaaataatggcagttaaagtaggtgatgccatgattatttttggtactttaatccattaggtgaaatttcatggtgcagtaataccactgttgttgtgttcctacttttgtggtaaatgtgggtgtttaaaaaattgtttctccaactcctttgagggtgttctgtgtagaggtttcttattggtggcacattttctatctctttggaaacatgagtgtatgaagtgtgcatgttgattgcatttttggcatgatgcattaacatgttttaaacttcaggcccttctactgccaaggtgagttcaggctgggcggctgcacccctgggagcagggcagtgctgcactgagccaggcgggagctggaagaagacgcagcacactggg |
| Luc-miR-744-B | agttgtactaactgaagattgaggcactggaataactaatgttgggtaagatagggtaagaactttttcgcaataaggtttgcagctcttgggattctcacttgataatgctgtttggataaaccatcatccaagtttgaataagatcttatatttagaaatgaaaactgatctgcagaattagtttaatcaataaatgggaatttttaaaaatcttcttggcaatttgtttttatgaatcccgttgaagctgtgtctattgactacctaaatctagatcattcttaatccagtgtgtaaaaaaccaggatgacacaaatgaaaaacttcaaaaacctggaggtcagactattttagtaatttagaaaacatttttcccacacattaatcagtactaaaagaaaaaagttaaaacctatttaaaatgtggaaaaattgcttcccaatattttaacagagagagactgagaacacacagcattgagtcatcaggaaaactgaagatctcccctgaacaacactgggatttcactgcagaggacttgaaagaccttggagaaattggacgaggagcttatggttctgtcaacaaaatggtccacaaaccaagtgggcaaataatggcagttaaagtaggtgatgccatgattatttttggtactttaatccattaggtgaaatttcatggtgcagtaataccactgttgttgtgttcctacttttgtggtaaatgtgggtgtttaaaaaattgtttctccaactcctttgagggtgttctgtgtagaggtttcttattggtggcacattttctatctctttggaaacatgagtgtatgaagtgtgcatgttgattgcatttttggcatgatgcattaacatgttttaaacttcaggcccttctactgccaaggtgagttcaggctgggcggctgcacccctgggagcagggcagtgctgcactgagccaggcgggagctggaagaagacgcagcacactggg |
| Luc-miR-744-C | AAGATCTCCCCTGAACAACACTGGGATTTCACTGCAGAGGACTTGAAAGACCTTGGAGAAATTGGACGAGGAGCTTATGGTTCTGTCAACAAAATGGTCCACAAACCAAGTGGGCAAATAATGGCAGTTAAAGTAGGTGATGCCATGATTA**TTTTTGG**TACTTTAATCCATTAGGTGAAATTTCATGGTGCAGTAATACCACTGTTGTTGTGTTCCTACTTTTGTGGTAAATGTGGGTGTTTAAAAAATTGTTTCTCCAACTCCTTTGAGGGTGTTCTGTGTAGAGGTTTCTTATTGGTGGCACATTTTCTATCTCTTTGGAAACATGAGTGTATGAAGTGTGCATGTTGATTGCATTTTTGGCATGATGCATTAACATGTTTTAAACTTCAGGCCCTTCTACTGCCAAGGTGAGTTCAGGCTGGGCGGCTGCACCCCTGGGAGCAGGGCAGTGCTGCACTGAGCCAGGCGGGAGCTGGAAGAAGACGCAGCACACTGGG |
| Luc-miR-744-C^mut^ | AAGATCTCCCCTGAACAACACTGGGATTTCACTGCAGAGGACTTGAAAGACCTTGGAGAAATTGGACGAGGAGCTTATGGTTCTGTCAACAAAATGGTCCACAAACCAAGTGGGCAAATAATGGCAGTTAAAGTAGGTGATGCCATGATTA**AAAAACC**TACTTTAATCCATTAGGTGAAATTTCATGGTGCAGTAATACCACTGTTGTTGTGTTCCTACTTTTGTGGTAAATGTGGGTGTTTAAAAAATTGTTTCTCCAACTCCTTTGAGGGTGTTCTGTGTAGAGGTTTCTTATTGGTGGCACATTTTCTATCTCTTTGGAAACATGAGTGTATGAAGTGTGCATGTTGATTGCATTTTTGGCATGATGCATTAACATGTTTTAAACTTCAGGCCCTTCTACTGCCAAGGTGAGTTCAGGCTGGGCGGCTGCACCCCTGGGAGCAGGGCAGTGCTGCACTGAGCCAGGCGGGAGCTGGAAGAAGACGCAGCACACTGGG |
